# Selective tracking of FFAR3-expressing neurons supports receptor coupling to N-type calcium channels in mouse sympathetic neurons

**DOI:** 10.1038/s41598-018-35690-z

**Published:** 2018-11-26

**Authors:** Claudia Colina, Henry L. Puhl, Stephen R. Ikeda

**Affiliations:** 1 National Institute of Neurological Disorders and Stroke, National Institutes of Health (NIH), Bethesda, Maryland 20892-9411 USA; 2 Section on Transmitter Signaling, Laboratory of Molecular Physiology, National Institute on Alcohol Abuse and Alcoholism, NIH, Bethesda, Maryland 20892-9411 USA

## Abstract

Activation of short-chain free fatty acid receptors 3 (FFAR3) has been suggested to promote sympathetic outflow in postganglionic sympathetic neurons or hamper it by a negative coupling to N-type calcium (Ca_V_2.2) channels. Heterogeneity of FFAR3 expression in sympathetic neurons, however, renders single neurons studies extremely time-consuming in wild-type mice. Previous studies demonstrated large variability of the degree of Ca_V_2.2 channel inhibition by FFAR3 in a global population of rat sympathetic neurons. Therefore, we focused on a small subpopulation of mouse sympathetic neurons using an FFAR3 antibody and an *Ffar3* reporter mouse to perform immunofluorescent and electrophysiological studies. Whole-cell patch-clamp recordings of identified FFAR3-expressing neurons from reporter mice revealed a 2.5-fold decrease in the Ca_V_2.2-FFAR3 inhibitory coupling variability and 1.5-fold increase in the mean I_Ca_^2+^ inhibition, when compared with unlabeled neurons from wild-type mice. Further, we found that the ablation of *Ffar3* gene expression in two knockout mouse models led to a complete loss-of-function. Subpopulations of sympathetic neurons are associated with discrete functional pathways. However, little is known about the neural pathways of the FFAR3-expressing subpopulation. Our data indicate that FFAR3 is expressed primarily in neurons with a vasoconstrictor phenotype. Thus, fine-tuning of chemically-coded neurotransmitters may accomplish an adequate outcome.

## Introduction

Free fatty acid receptors (FFAR1–3) were cloned in the search for novel receptors^1^ and classified as orphan proteins in 1997. A few years later, short chained fatty acids (SCFAs) were identified as agonists by two different laboratories^2,3^. Free fatty acid receptors are included in a branch of nucleotide receptors, within the most recent evolutionarily arm of the phylogenetic tree (Class A) of G protein-coupled receptors (GPCR). FFAR1–3 share approximately 30–40% identity and have a limited ligand specificity that differs between species. Among FFARs, FFAR3 is predominantly activated by acetate, propionate and butyrate. These ligands originate mostly as by-products of undigested carbohydrate fermentation by anaerobic bacteria in the lower gut^2^ and following ethanol ingestion and subsequent metabolism. FFAR3 is found on intestinal enteroendocrine cells, enteric neural plexuses^4–8^, pancreatic islets^9–11^, vascular endothelium^12^, adipose tissue^13^, antigen-presenting cells^14^, gastric brush cells^15^ and the sympathetic nervous system (SNS)^16–18^. FFAR3 has been implicated in inflammation^19^, allergic airway diseases^14^, metabolic disorders^6,11,20,21^, diabetes^11^, immune function^14,22^ and in promoting sympathetic tone^17,23^.

The canonical GPCR signaling pathway has been well established and entails ligand binding to the receptor and a subsequent exchange of GDP for GTP on the Gα−subunit of the heterotrimeric Gαβγ protein. Subsequently, Gα-GTP dissociates from Gβγ allowing each moiety to modulate discrete effector proteins. An example of such modulation is the association of dimeric Gβγ with the N-type calcium channels to negatively modulate calcium currents^24–28^. This inhibition (I_Ca_^2+^ inhibition) has unique characteristics, such as being membrane delimited and having slow kinetics in the activation phase at moderate membrane potentials^28^. However, after a strong depolarization, a temporary relief of inhibition occurs and the kinetics are faster^28^. An overnight incubation of rat FFAR3-expressing neurons with *Bordetella pertussis* toxin (PTX) abolished this distinctive inhibition by uncoupling the receptor from the signaling pathway^18^. FFAR3 thus couples specifically to the G_i/o_ family of the heterotrimeric family of G-proteins^2,3,29^ and consequently can decrease cAMP production^17^.

FFAR3 is expressed in a subpopulation of sympathetic neurons, thus electrophysiological studies with dissociated neurons are challenging due to the substantial cell-to-cell response variability^18^. In this study, recording from identified neurons isolated from an established reporter mouse line resulted in 2.5-fold decrease in response variability and a 1.5-fold increase in the mean I_Ca_^2+^ inhibition. Our findings appear to be substantiated by the fact that *Ffar3* gene loss (nullizygote) led to a complete ablation of SCFA induced Ca^2+^-channel inhibition in mice sympathetic neurons, confirming the requirement for FFAR3 in recorded responses.

The superior cervical ganglia (SCG) and the celiac-mesenteric ganglia (CSMG) are part of the sympathetic subdivision of the autonomic nervous system (ANS). Both ganglia contain discrete neuronal subpopulations with a distinctive “neurochemical code” that selectively projects to functionally heterogeneous peripheral targets. This provides a fine-tune control of different sympathetic pathways. SCG project vasoconstrictor and secretomotor/sudomotor fibers to the head and neck, secretory fibers to salivary glands, dilator papillae, eyelid/orbitalis non-striated muscle, pilomotor fibers to arrector pili muscles, and vasoconstrictor fibers to the heart and lung. Likewise, CSMG vasoconstrictor, vasodilator, secretomotor/sudomotor and pilomotor neurons innervate a variety of visceral structures and mesenteric and renal vessels^30^. The main neurotransmitter in these sympathetic neurons is norepinephrine (NE), either in isolation or with other neurotransmitters such as neuropeptide Y (NPY) and adenosine triphosphate (ATP), among others. However, exceptions to this general rule are cholinergic sympathetic nerve endings innervating sweat glands^31^, and non-cholinergic/non-noradrenergic vasodilator neurons supplying some skeletal muscle vessels^32,33^, and skin blood vessels in rodents.

Currently, little is known about the “chemical coding” of FFAR3-expressing sympathetic neurons. In this study, we found that the vast majority of FFAR3-expressing neurons possess a vasoconstrictor phenotype. These results represent a first step toward enhancing our understanding about this small subpopulation of FFAR3-expressing sympathetic neurons.

## Results

### *Ffar3* gene is expressed in mouse sympathetic ganglia and does not change during development

We began by assessing the levels of *Ffar3* mRNA transcript within the mouse SCG and CSMG. Unlike previous studies on rat SCG^18^ or young adult mice (PND 49)^17^, we used a custom-made TaqMan primers-probe set designed to overlap an exon-exon junction, reducing the possibility of false positive qPCR reactions. A screening of 24 different mouse tissues showed that SCG and CSMG ganglia have the highest levels of *Ffar3* mRNA transcripts (Supplementary Fig. 1a). These results are qualitatively consistent with previous attempts to establish *Ffar3* gene expression in mice^17^. Additionally, a low number of transcripts were present in other tissues, although there was some biological variability.

Even though rodents undergo a great deal of remodeling and development during the early postnatal period, including the death of one-third of rat SCG cells by apoptosis over the first postnatal week^34^, we did not observe substantial dynamic changes in *Ffar3* mRNA levels in either ganglia from birth to PND21, or in 1 year old animals (Supplementary Fig. 1b).

### Sodium propionate induced variable I_Ca_^2+^ modulation in neurons from wild-type mice but not from FFAR3 knockout mice

**Figure 1:**
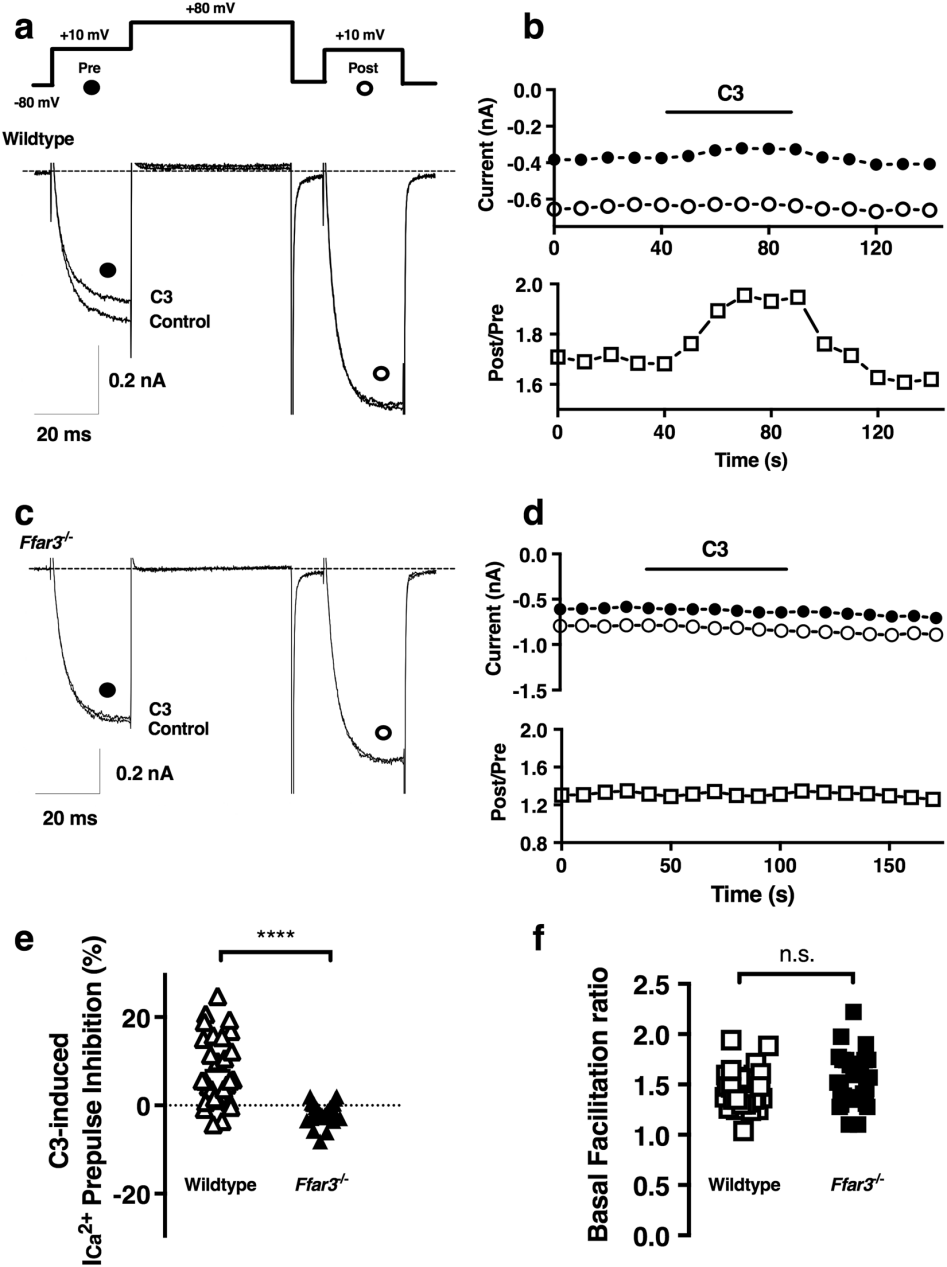
Sodium propionate-induced I_Ca_^2+^ modulation in wild-type acutely dissociated CSMG neurons with a loss-of-function in knockout mice. (**a**) Double-pulse voltage protocol evoked every 10 s (see inset in **a**) consisting of two identical test pulses (+10 mV for 25 ms from a holding potential of −80 mV), separated by a large depolarizing conditioning pulse to +80 mV for 50 ms. Representative time courses of current inhibition and superimposed current traces in the absence (control) and the presence of 1 mM sodium propionate (C3), obtained from wild-type (*Ffar3*^+/+^, **a**) and *Ffar3* knockout mouse (*Ffar3*^−/−^, **c**) from CSMG dissociated neurons. Calibration: y-axis 0.2 nA and x-axis 20 ms for both. I_Ca_^2+^ amplitudes in a and c, were measured isochronally at 10 ms in prepulses (filled circles, **a**–**d**) and postpulses (open circles, **a**–**d**) and values were used in b and d plots. Likewise, time course of facilitation ratio (Post/Pre, open squares in lower panel in b and d) were plotted. (**e**) Comparison of the percentage of I_Ca_^2+^ inhibition between wild-type (white triangles) and *Ffar3*^−/−^ (black triangles), from acutely dissociated CSMG neurons. Two-tailed Welch’s *t*-test was used to assess if the wild-type and *Ffar3*^−/−^ had equal means. Both populations were statistically different using a two-tailed Welch’s *t*-test (*t*(33.1) = 6.2, with α = 0.1, 90% CI: 6.9–12.0, n_1_ = n_2_ = 29 and *p* < 0.0001). (**f**) Basal facilitation ratio plot between wild-type (white square) and *Ffar3*^−/−^ (black square) from CSMG dissociated neurons, showed not significant difference when a two-tailed *t*-test. *t*(56) = 1.4, with α = 0.1, 90% CI: −0.19–0.02, n_1_ = n_2_ = 29 and *p* = 0.17. Four *Ffar3*^+/+^, and three *Ffar3*^−/−^ animals were used to generate the data shown in figures. Dotted lines represent zero current in A and zero % I_Ca_^2+^ inhibition in (**e**).

Won, *et al*. (2013) reported that activation of FFAR3 inhibited Ca_V_2.2 currents in sympathetic neurons isolated from Wistar rats. The inhibition was both voltage-dependent (VD) and sensitive to pretreatment with PTX^18^. These results were corroborated by heterologous expression of the protein^18^. To extend these findings to mice, we performed voltage clamp experiments with CSMG neurons dissociated from wild-type mice. We measured Ca^2+^ currents using the voltage protocol illustrated in Fig. 1a (top), elicited every 10 s. The protocol consists of holding the membrane potential at −80 mV and applying a set of four voltage steps. The first test pulse to +10 mV (Prepulse or Pre, black circles) assesses baseline inhibition of the Ca^2+^ currents. The second strong depolarizing step to +80 mV is expected to promote the release of the Gβγ dimer from its calcium channel binding pocket. After channels are briefly closed with a voltage step to −80 mV, they are allowed to open again by a Postpulse to +10 mV (Postpulse or Post, white circles). VD inhibition of the Ca^2+^ currents is quantified by the facilitation ratio, the postpulse steady-state Ca^2+^ currents amplitude (Equation 2) divided by the prepulse current amplitude (Post/Pre). The facilitation ratio (Fig. 1b, lower panel), provides a surrogate for degree of Ca^2+^ channel modulation mediated by the Gβγ limb of the signaling pathway; a larger facilitation ratio indicates a greater I_Ca_^2+^ inhibition by this mechanism. Figure 1a (bottom) shows representative current recordings in response to the voltage protocol described above. Ca^2+^ current amplitudes after the strong depolarization step to +80 mV (Fig. 1a, open circle/Post) are larger than the amplitude in the Prepulse (Fig. 1a, black circle/Pre), indicating recovery from inhibition due to the release of the Gβγ dimer from the channels during the +80 mV step.

The current recording labeled C3 represents the response to application of 1 mM sodium propionate. As previously observed in rat sympathetic neurons, propionate produced a voltage-dependent inhibition of the prepulse Ca^2+^ current (Fig. 1a). Figure 1b shows the time course of the entire experiment. In the top panel the prepulse (black circles) and the postpulse Ca^2+^ currents amplitude (white circles) are plotted, and in the bottom panel, the facilitation ratio (white squares) is shown. This CSMG neuron showed a substantial increase in the I_Ca_^2+^ inhibition in the presence of 1 mM C3 (Upper panel in Fig. 1b); rising to an ~15% facilitation ratio (Lower panel in Fig. 1b). Figure 1e (white triangles) shows there is substantial cell-to-cell variation in the response to 1 mM C3, as previously reported in rats^18^. However, when acutely dissociated CSMG neurons from FFAR3-knockout mice (*Ffar3*^−/−^) were used, propionate application no longer produced inhibition (Fig. 1c–e).

Further, the basal facilitation ratios prior to the exposure to C3 were similar in neurons from wild-type and knockout mice (Fig. 1f). This might suggest little to no constitutive activation of the FFAR3 voltage-dependent inhibitory pathway in accordance with previous *in vitro* studies^35^. Likewise, no change in the I_Ca_^2+^ density was observed between wild-type and *Ffar3* deficient mice (Supplementary Fig. 2).

### *Ffar3*^+/*mRFP*^ mice recapitulate endogenous expression patterns in CSMG and SCG

**Figure 2:**
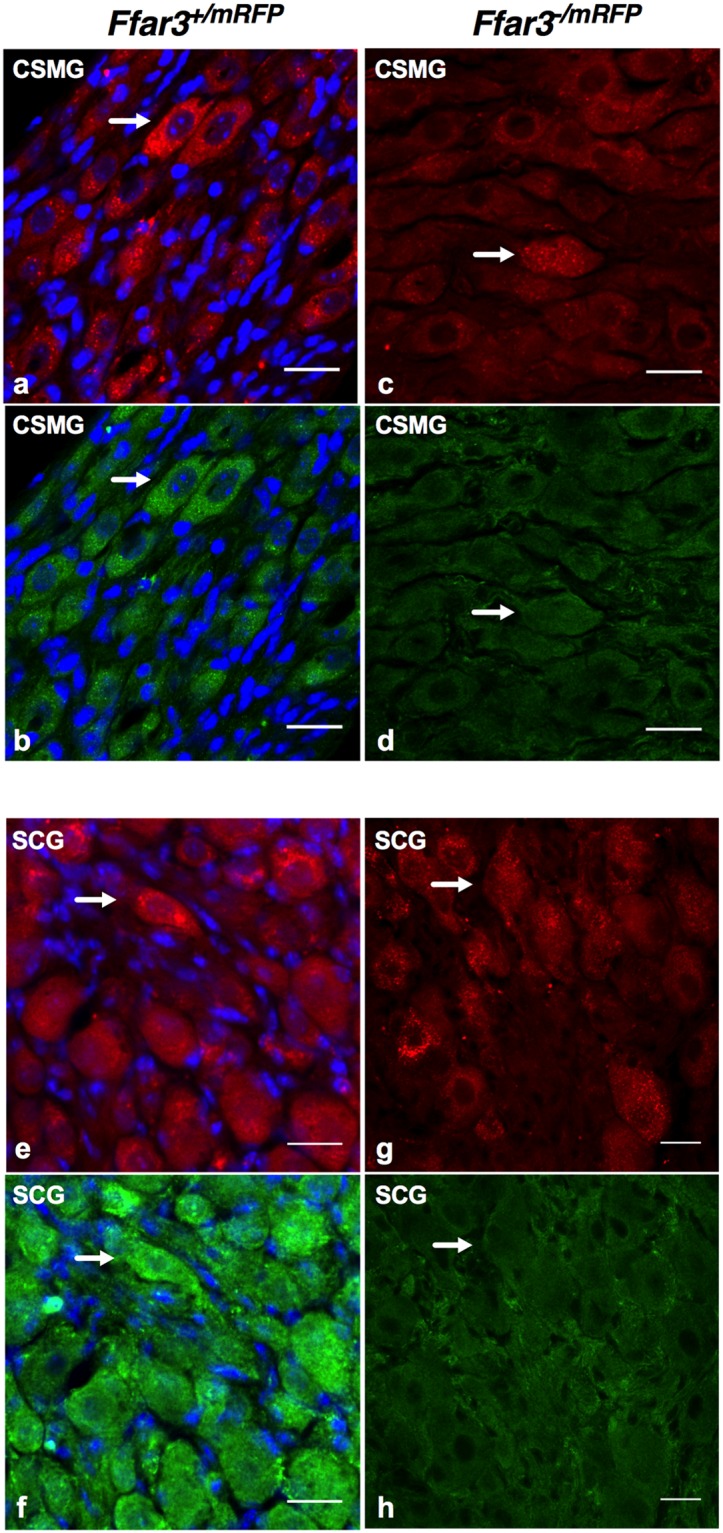
Cryosections of mice celiac mesenteric ganglia and superior cervical ganglia FFAR3-labelled, showing association of the Ffar3-reporter hemizygous/nullizygous mice with FFAR3 antibody. Representative fluorescent IF images of three repetitions with CSMG/SCG cryosections from hemizygous *Ffar3*^+/*mRFP*^ (CSMG: **a**,**b**, SCG: **e**,**f**) and nullizygous *Ffar3*^*−/mRFP*^ (CSMG: **c**,**d**, SCG: **e**,**f**) reporter mice. Left upper panel: Direct mRFP (red, **a**) fluorescence detection in a CSMG slice counterstained with anti-FFAR3 antibody (green, **b**) and DAPI (blue, (**a**,**b**) on hemizygous *Ffar3*^+/*mRFP*^ reporter. Right upper panel: Direct mRFP (red, **c**) fluorescence detection in CSMG slice counterstained with anti-FFAR3 antibody (green, **d**) from nullizygous *Ffar3*^*−/mRFP*^ reporter mice. Lower left Panel: Direct mRFP fluorescence (red, **e**) detection in SCG slices counterstained with Anti-FFAR3 antibody (green, **f**) and DAPI (blue **e**,**f**) from hemizygous *Ffar3*^+/*mRFP*^ reporter. Lower right panel: Direct mRFP (red, **g**) fluorescence detection in SCG slices counterstained with anti-FFAR3 antibody (green, **h**) from nullizygous *Ffar3*^*−/mRFP*^ reporter mice. Note that in both ganglia, mRFP and FFAR3 antibody fluorescence (white arrow) are restricted to a small subpopulation of neurons, when compared to the visible nuclei (total cell types within each ganglion e.g. SIF-like cells, Schwann cells ensheathed nerve fibers, satellite cells and neurons). Scale bars in all images are 20 μm.

We hypothesized that the large response variability in wild-type mice was due to heterogeneity of FFAR3 expression in CSMG neurons. To investigate this possibility, we used a transgenic FFAR3 reporter mouse model (*Ffar3*^+/*mRFP*^)^16^ that expresses both FFAR3 and monomeric red fluorescent protein (mRFP), under the control of the mouse *Ffar3* promoter. First, we counter-labeled mRFP-positive cells with a rabbit polyclonal FFAR3 antibody^8^ in order to examine labeling concurrence in the reporter mouse. We did not study the degree of spatial colocalization between FFAR3 antibody and mRFP fluorescence as the antibody is mostly membrane located, whereas mRFP was primarily located in the cytoplasm. Figure 2a,b shows one representative images from a longitudinal cryosection of CSMG with mRFP/DAPI and anti-FFAR3/DAPI fluorescence, respectively. With four randomly selected CSMG images, we found that 21.7 ± 2.7% of DAPI positive cells were mRFP-positive. The majority of the cells showed concurrence with FFAR3 positive cells, only a small number (2.1%) of FFAR3 positive cells were not mRFP positive. These observations suggest that a minor subpopulation of sympathetic neurons express FFAR3 in the mouse CSMG.

We also crossed the reporter and knockout mouse strains to obtain a *Ffar3*^*−/mRFP*^mouse to perform additional experiments. Neurons from the *Ffar3*^*−/mRFP*^mouse lacked FFAR3 immunoreactivity in CSMG sections (Fig. 2c,d). The lack of both immunofluorescences in dorsal root ganglion (DRG) sections from these animals (Supplementary Fig. 3) are provided as negative control.

**Figure 3:**
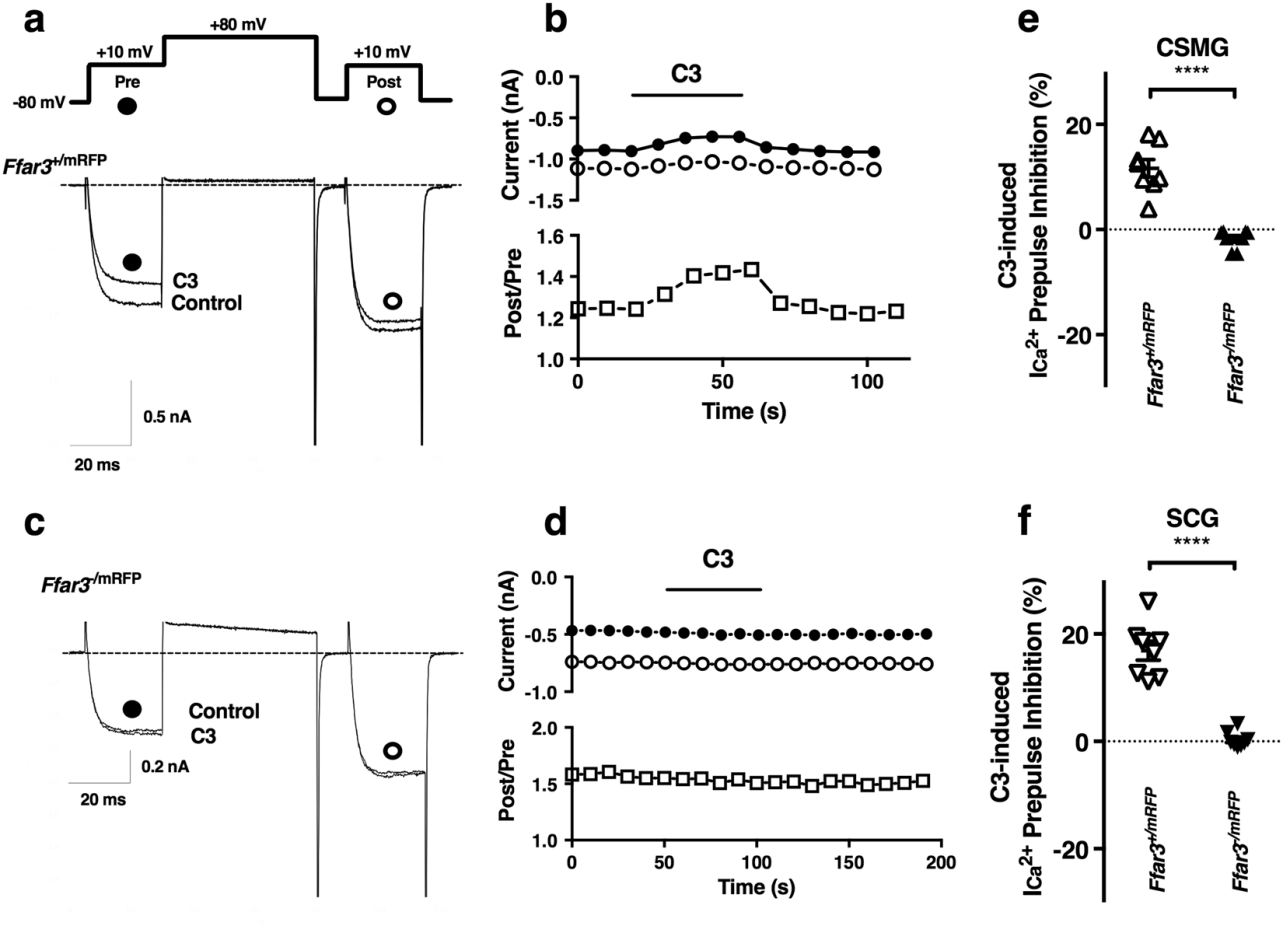
Sodium propionate-induced I_Ca_^2+^ modulation in a small subpopulation of FFAR3-expressing CSMG neurons with a loss-of-function in the nullizygous mice. (**a**) Double-pulse voltage protocol evoked (see inset in **a**) and acquired as described in Fig. 1. Representative time courses and superimposed I_Ca_^2+^ traces in the absence (control) and the presence of 1 mM C3, obtained from *Ffar3*^+/*mRFP*^ (**a**) and *Ffar3*^*−/mRFP*^ (**c**) from acutely dissociated CSMG neurons. Calibration: y-axis 0.5 nA (lower panel in a), 0.2 (lower panel in c) and x-axis 20 ms for both. I_Ca_^2+^ amplitudes were measured isochronally at 10 ms as in Fig. 1 and values were used in (**b**,**d**) plots. The amplitude of currents generated by prepulses (black circles **a**–**d**) and postpulses (white circles, **a**–**d**) were used to calculate % of I_Ca_^2+^ inhibition and plotted in e. Lower panels in b and d show facilitation ratio (Post/Pre, white squares in, **b**,**d**). (**e**) Comparison of the % of I_Ca_^2+^ inhibition induced by 1 mM C3 between *Ffar3*^+/mRFP^ (white up-pointing triangles) and *Ffar3*^*−/mRFP*^ (up-pointing black triangles), from acutely CSMG dissociated neurons. (**f**) Comparison of the % of I_Ca_^2+^ inhibition induced by 1 mM C3 between *Ffar3*^+/mRFP^ (down-pointing white triangles) and *Ffar3*^*−/mRFP*^ (down-pointing black triangles), from acutely SCG dissociated neurons. Two-tailed Welch’s *t*-test was used to determine if the difference in means of the I_Ca_^2+^ inhibition between hemizygous (*Ffar3*^+/*mRFP*^) and nullizyogous (*Ffar3*^*−/mRFP*^) was equal zero. Both populations were statistically different (CSMG: *t*(8.4) = 7.5, with α = 0.01, 99% CI: 7.2–18.7, n_1_ = n_2_ = 8 and *p* < 0.0001; SCG: *t*(7.4) = 9.5, with α = 0.01, CI: 10.9–23.1, n_1_ = n_2_ = 8 and *p* < 0.0001). Three *Ffar3*^+/*mRFP*^ animals and four *Ffar3*^*−/mRFP*^ animals were used to generate data shown in figures. Dotted lines represent zero current in a, b and zero % I_Ca_^2+^ inhibition in (**e**,**f**).

These results indicated that the reporter mouse was a suitable model for live cell identification of FFAR3-expressing neurons. Figure 3a–d shows electrophysiological experiment similar to those shown in Fig. 1, but with recordings for identified FFAR3-expressing neurons from acutely dissociated CSMG cultures. In eight experiments with this preparation, we did not observe a subpopulation of neurons with a negligible response to C3 application (Fig. 3e; open triangles). Consequently, there was a 2.5-fold decrease in the coefficient of variation with *Ffar3*^+/*mRFP*^ mouse CSMG dissociated neurons as compared to those from wild-type mice (40 *vs* 100%, respectively). These results confirm that the cell-to-cell variability in wild-type mice was because not all neurons in the CSMG express FFAR3. There was lack of response to C3 in fluorescent CSMG neurons from the *Ffar3* ^*−/mRFP*^mouse model (Fig. 3c–e), consistent with *Ffar3* ablation as previously observed with CSMG neurons from the *Ffar3*^−/−^ mouse model. Thus, suggesting that the I_Ca_^2+^ inhibition produced by C3 application arose solely from FFAR3.

Since FFAR3 expresses abundantly in the SCG, we performed immunocytochemistry and electrophysiology with this tissue. Similar to CSMG, we observed that a subpopulation of DAPI positive cells (17.2 ± 1.6%) were mRFP-positive (Fig. 2e). From this subpopulation, the majority of cells showed concurrence with FFAR3 immunoreactive (IR) labeling (Fig. 2f). Only 1.9% of FFAR3 positive cells were mRFP negative. SCG cryosections from *Ffar3*^*−/mRFP*^mice lack FFAR3 immunoreactivity (Fig. 2g,h). All fluorescent (mRFP) SCG neurons displayed a C3-induced Ca^2+^ channel inhibition (Fig. 3f) as was observed in CSMG (Fig. 3e). A slightly higher mean I_Ca_^2+^ inhibition (16.9 ± 1.76%; n = 8) was observed as compared to CSMG neurons (11.64 ± 1.65%; n = 8), consistent with a marginally higher levels of FFAR3 transcripts observed in SCG (Supplementary Fig. 1a–c).

Interestingly, the level of I_Ca_^2+^ modulation was not impaired by the presence of a single null allele. FFAR3 transcript levels in the hemizygous mouse, that has one *Ffar3* allele (*Ffar3*^+/*mRFP*^, Supplementary Fig. 1c), were comparable to *Ffar3* mRNA levels in wild-type mice (Supplementary Fig. 1b). This might be explained by an autosomal dosage compensation, which has been reported in other organisms^36–39^.

### Phenotypic identification of FFAR3-IR vasoconstrictor sympathetic neurons

**Figure 4:**
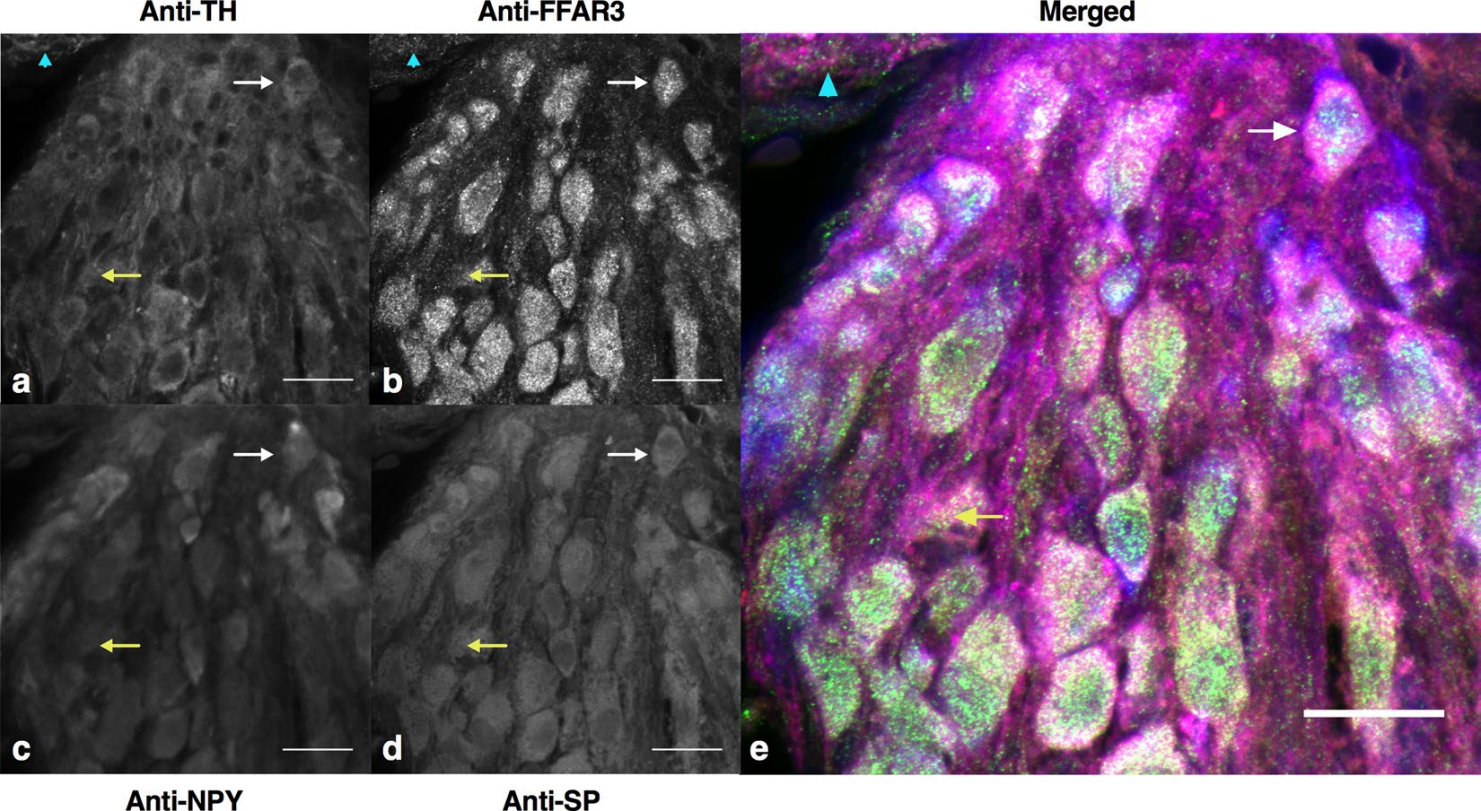
Cryosections of mice celiac-mesenteric ganglia quadruple-labelled to show chemical coding of postganglionic neurons and FFAR3-expressing neurons. Quadruple-staining of CSMG sections were performed with Anti-Tyrosine hydroxylase (Anti-TH, **a**) Anti-FFAR3 (**b**) Anti-Neuropeptide Y (Anti-NPY, **c**) and Anti-Substance P (Anti-SP, **d**). White arrow indicates a representative neuron SP-, TH-, NPY- and FFAR3-IR and presumed to be a vasoconstrictor neuron. Yellow arrow indicates a representative neuron SP-, TH-IR and NPY-, FFAR3-IN. Cyan arrowhead indicates a TH-IR fiber surrounded by FFAR3 punctuate immunoreactivity (**a**,**b**,**e**). Labeling was repeated twice and using four wild-type animals on each replicate. Scale bars in all images are 20 μm.

**Figure 5:**
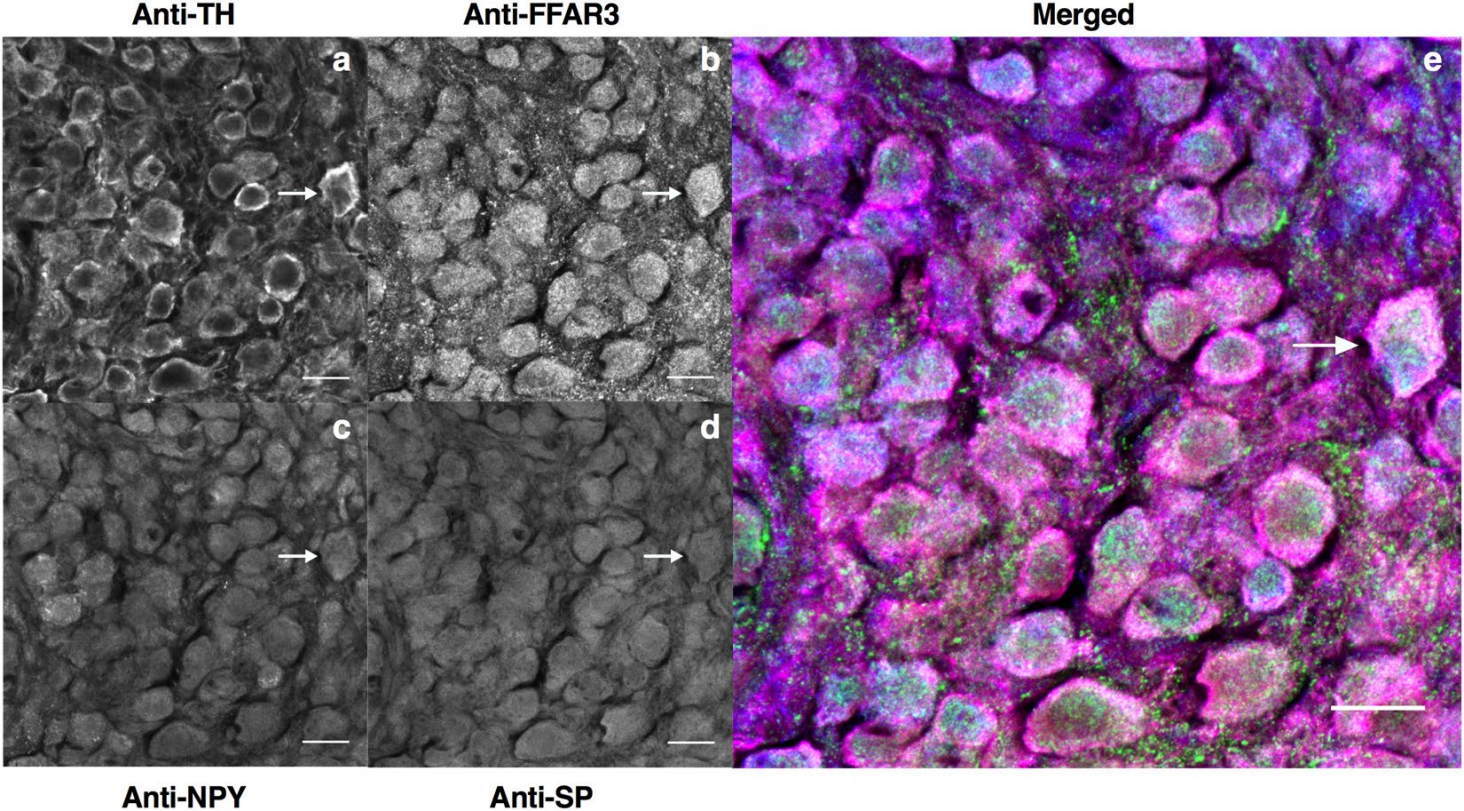
Cryosections of mice superior cervical ganglia quadruple-labelled to show chemical coding of postganglionic neurons and FFAR3-expressing neurons. Quadruple-staining of SCG sections were performed with Anti-Tyrosine hydroxylase (Anti-TH, **a**) Anti-FFAR3 (**b**) Anti-Neuropeptide Y (Anti-NPY, **c**) and Anti-Substance P (Anti-SP, **d**). White arrow indicates a representative neuron SP-, TH-, NPY- and FFAR3- IR and presumed to be a vasoconstrictor neuron. Labeling was repeated twice and using four wild-type animals on each replicate. Scale bars in all images are 20 μm.

Little is known about the “chemical coding” of sympathetic FFAR3-expressing neurons. To explore this categorization, we labeled CSMG and SCG cryosections with selected antibodies (Figs 4 and 5 and Supplementary Figs 4, 5). Using 5 randomly selected cryosections from CSMG and 4 from SCG, we visualized coincidence of calbindin-28K (Cb300), ATP-gated ionotropic P2X_2_ receptor (P2X_2_), neurofilament 200 kDa (αNF200) and FFAR3 immunoreactivity (Supplementary Figs 4, 5). Within the cell body compartment, most FFAR3-IR neurons from either ganglion were immunoreactive for P2X_2_ (Supplementary Figs 4a and 5a). It has been shown that the P2X receptor (a ligand-gated ion channel triggered by ATP) enhances NE release from sympathetic nerve endings^40–43^. Also, ATP and NE are co-released from synaptic vesicles in sympathetic nerves and evoke contraction on visceral and vascular smooth muscle^44–46^. The majority of rat sympathetic neurons/cells contains mainly the P2X_2_ isoform of the purinergic receptors and perhaps a heteromeric receptor containing P2X_2/3_^47^.

A cluster of P2X_2_-IR and FFAR3 immunonegative (IN) small diameter cells were consistently observed among SCG postganglionic neurons (yellow arrow, Supplementary Fig. 5). The distribution and size resemble small intensely fluorescent (SIF) cells (SIF-like cells), which were scarcely labeled by the FFAR3 antibody. Supplementary Fig. 4 shows a bundle of axons in which some were NF200-IR, indicating they were myelinated fibers more likely projecting from a subpopulation of DRG neurons^48^. Some myelinated fibers were also immunoreactive to Cb300, most likely derived from Schwann cells (orange arrowhead, Supplementary Fig. 4c–e). In some sections, these fibers are intermingled between the cells (Supplementary Fig. 5c,e). The intensity of Cb-300 immunofluorescence (IF) in sympathetic postganglionic neurons was variable. Some neurons had an intense immunofluorescence (SCG, Supplementary Fig. 5d), while others were scarcely above the background level present in the ganglia (CSMG, Supplementary Fig. 4d), as previously reported for this ganglion^49–51^. A quantitative analysis of these cell bodies showed that 98 out 150 CSMG neurons were FFAR3-IR (Supplementary Fig. 4b) and 93.9 ± 2.4% of these cells were also P2X_2_-IR (Supplementary Fig. 4a) and rarely Cb300-IR (Supplementary Fig. 4d). In contrast, 133 out 163 SCG neurons were FFAR3-IR (Supplementary Fig. 5b), 99.3 ± 0.8% P2X_2_-IR (Supplementary Fig. 5a) and 70.7 ± 4% Cb300-IR (Supplementary Fig. 5d).

**Figure 6:**
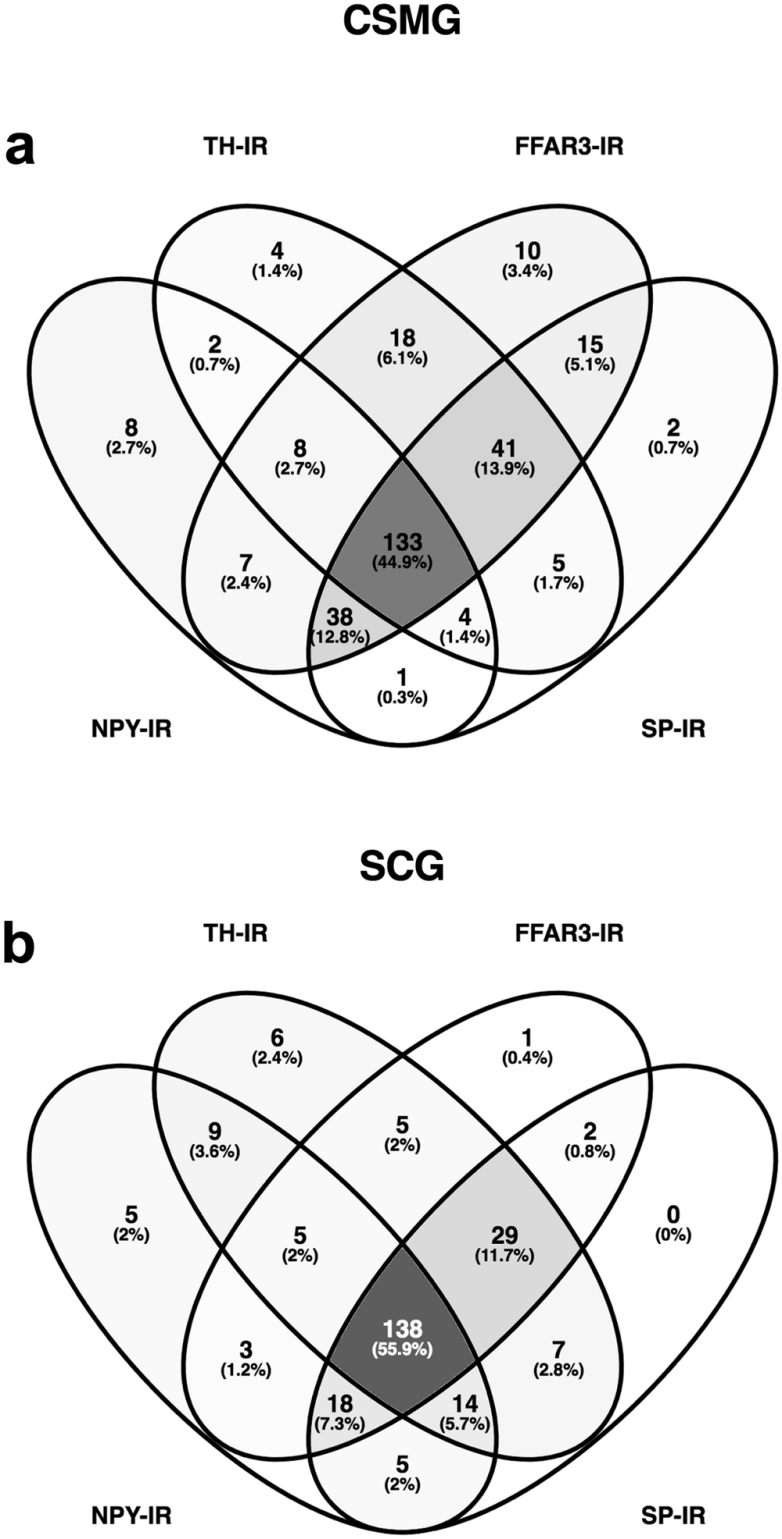
Chemical coding of FFAR3-expressing neurons. Venn diagrams summarizing the frequencies and overlap of TH, NPY, SP and FFAR3 positive neurons within CSMG (**a**) and SCG (**b**) images. Percentages were calculated from the total number of labelled neurons in each ganglion (CSMG: 296 cells and SCG 247 cells) from randomly selected cryosections. Note that most of sympathetic neurons enriched with FFAR3 are noradrenergic vasoconstrictor neurons.

In another set of cryosections, we used tyrosine hydroxylase (TH), neuropeptide Y (NPY) and substance P (SP) antibodies to study their concurrence with FFAR3. Figures 4 and 5 show representative cryosections from CSMG and SCG, respectively. Using four randomly selected images from CSMG and five from SCG for analysis, we scored 296 neurons from the former and 247 neurons from the latter. Each neuron was scored either positive (1) or negative (0) for the four markers. Figure 6a,b show the Venn diagrams for both ganglia. In general, they are quantitatively similar. About 70% of neurons are immunoreactive to TH and FFAR3, which makes them almost certainly noradrenergic cells. About 50% of this group are also NPY/SP positive, which is consistent with previously reported studies in which 2/3 of the noradrenergic sympathetic neurons in several species express NPY^52–54^. These types of neurons are vasoconstrictor in function and represent the vast majority of sympathetic neurons^55,56^.

## Discussion

Through the combined use of electrophysiological and immunofluorescent approaches, together with a fluorescent reporter mouse strain, we have examined FFAR3-expressing neurons in two mouse sympathetic ganglia. The monoallelic presence of mRFP allowed a targeted functional study of FFAR3, showing that activation of this receptor is negatively coupled to Ca_V_2.2 in a small subpopulation of vasoconstrictor sympathetic neurons. A key to unraveling how sympathetic neurons influence target tissues is understanding which subpopulation is enriched with the protein of interest and its functional pathway. In this study, we examined these questions.

First, we examined the levels of FFAR3 transcripts in sympathetic ganglia alongside a battery of mouse tissues, as well as in the transgenic mouse strain. We found abundant levels of FFAR3 transcript in both SCG and CSMG, which agrees with previous findings^16–18^. Wright, *et al*., 1983 found that around one-third of rodent’s SCG cells die by apoptosis within the first postnatal week^34^. However, we did not observe changes in the transcript levels of FFAR3 in SCG, or CSMG. The reason for this apparent contradiction is still not entirely clear, but it could be argued that both approaches (qPCR in this study) and electron microscopy^34^ suffer from some limitations (e.g., availability of suitable reference genes that are unaffected by animal development and between tissues, lack of distinction between SIF-like cells *vs* neurons^34^, cell specific death of FFAR3-non expressing neurons, etc.).

A neglected area in the field has been functional studies with identifiable FFAR3-expressing neurons. Experiments with unlabeled mouse CSMG neurons studying the C3-induced ICa_2_^+^ inhibition showed a highly variable response (Fig. 1); a result similar to that described previously in rats^18^.

Tracking FFAR3 expression in neurons within SCG and CSMG through a combined use of an *Ffar3*-reporter mouse strain, an FFAR3 antibody and DAPI, indicated that the receptor is expressed in a small subpopulation of ganglionic cells. Consequently, any set of data derived from a global approach is poised to be highly complex and variable, as we showed when using acutely dissociated CSMG neurons from wild-type mice (Fig. 1). Taking advantage of a previously established reporter mouse^16^, we found a 1.5-fold increase in the ICa_2_^+^ inhibition (Wt~8% *vs* FFAR3^+/mRFP^~12%), by recording only from mRFP-positive CSMG neurons. Likewise, Ca_V_2.2-FFAR3 coupling was recorded with acutely dissociated mouse SCG neurons (SCG: ICa_2_^+^ inhibition ~17%). Most importantly, by using CSMG neurons from the reporter mouse^16^, the variability of the response was decreased (Fig. 3e,f). Further, when the receptor was genetically ablated in two mice strains^6,16^, a loss-of-function was observed in both ganglia. These results provide direct evidence supporting a functional link between Ca_V_2.2 channels and FFAR3 in mouse sympathetic neurons.

Previous studies in our laboratory demonstrated that the number of neurons exhibiting Ca_V_2.2-FFAR3 coupling in wild-type rats was low in SCG^18^. However, it appeared to gradually increase in other ganglia located more caudally like the stellate ganglia, CSMG and the major pelvic ganglia. Since we observed comparable levels of neurons with Ca_V_2.2-FFAR3 coupling between SCG and CSMG, this rostral-caudal gradient appears not to be present in mice. Further, CSMG from wild-type mice seem to have lower ICa_2_^+^ inhibition elicited by C3 as compared to wild-type rat. These results suggest that there might be a species-specific difference in FFAR3 expression within the sympathetic nervous system (e.g., cell size, protein density, etc.).

Activation of FFAR3 by short chain fatty acids produces an inhibition of Ca_V_2.2 channels in CSMG and SCG neurons (Figs 1 and 3). The functional consequences of this would depend on the location of these receptors. Even though we do not know if they are present at the axon terminal, a decrease of Ca_2_^+^ influx in this region would diminish neurotransmitter release. We do know, however, that FFAR3 is abundantly expressed at the soma of CSMG and SCG neurons (Figs 4 and 5).

There might be at least two important roles for these receptors at the soma. First, FFAR3-induced activation of Gβγ-PLCβ-MAPK signaling^17^, which may lead to profound physiological changes in sympathetic neurons. Second, a reduction in Ca^2+^ influx may influence the excitability of sympathetic neurons and also produce long-term physiological effects by influencing diverse Ca^2+^-dependent membrane proteins, intracellular calcium sources, kinases, protein translocation and gene expression. In mammalian sympathetic neurons, Ca^2+^ influx during the action potential produces an after-hyperpolarization (AHP), that may last a few milliseconds. A direct impairment in the Ca^2+^ influx though N-type calcium channels, and indirectly throughout a decrease in the Ca^2+^ induced Ca^2+^ release (CICR) from intracellular stores, has been associated with a marked decreased (~60%) of the AHP. This is due to less activation of the outward current through apamin-sensitive small-conductance Ca^2+^-activated potassium channels (SK type)^54,57^, which reduces spike-frequency adaptation and increases neuron excitability. However, the direct functional effect would rely on the close proximity of these two types of channels, which has not been directly demonstrated in mouse sympathetic neurons.

Despite important species differences, there is some discrete neurochemical coding in sympathetic pathways that has been previously described based on retrograde labeling, markers and electrophysiology^30,54,55,58,59^. It is widely accepted that the vast majority of mammalian sympathetic neurons can be divided by immunofluorescence on the basis of two markers (TH and NPY). To date, little is known about the neurochemical coding of FFAR3-expressing neurons. Multiple-labelling immunofluorescence in this study showed for the first time that only a subpopulation of sympathetic neurons express FFAR3. The largest FFAR3-expressing subpopulation of neurons from mouse SCG/CSMG, are noradrenergic neurons in nature (SCG ~83; CSMG ~93%). Previous studies show that TH/NPY containing neurons are likely a subgroup of vasoconstrictors neurons with a high presynaptic stimulus threshold^54^, slow postganglionic axonal conduction velocity^54^, and that comprise more than half of all ganglionic cells in guinea-pig lumbar sympathetic chain^55^. We found that 67% and 65% FFAR3-expressing neurons are noradrenergic vasoconstrictors in SCG and CSMG, respectively.

A small subpopulation of non-noradrenergic sympathetic neurons in rat^32^, mouse^32,60^ guinea pig^52,55,61^, and rabbit^59,60^, species has been shown to innervate a variety of target tissues. In fact, we have also found a small subpopulation of neurons enriched with FFAR3 that are non-noradrenergic (SCG ~12; CSMG ~26%).

Likewise, TH-IR neurons lacking NPY are likely pilomotor/secretomotor neurons and were classified as low-threshold secretomotor and high threshold pilomotor neurons previously by electrophysiological studies^54^. Our data show that 16 and 28% of FFAR3-expressing neurons are pilomotor/secretomor in SCG and CSMG, respectively. The plurichemical nature of sympathetic terminals^62–65^ has been well established. The consequences of sympathetic nerve activation depend not only on the vesicle content, but also on the intensity and pattern of activation, as well as the combination of receptors present at target regions, and the postsynaptic effect that each neurotransmitter exerts on each other as well as on their own receptors located at the cell bodies^46^. Our data suggest that in both sympathetic ganglia the large majority of FFAR3 positive neurons might be plurichemical in nature. Indeed, many were positive to TH, NPY and SP antibodies (Fig. 6).

In summary, we show that the N-type calcium channel is inhibited by FFAR3 activation in acutely dissociated mouse sympathetic neurons, a response that is abolished in two different *Ffar3* knockout mice. Likewise, we have shown that FFAR3 is expressed in a subpopulation of vasoconstrictor sympathetic neurons and in to a lesser extent in other cell populations, unveiling a heterogeneity previously unknown. An extensive functional study is necessary to determine the implication of such heterogeneity. Nevertheless, it is likely that the presence or absence of a particular neurotransmitter in FFAR3-expressing neurons, in particular vascular beds, will lead to a qualitative outcome at the target tissue.

## Methods

### Animal Models

*Ffar3* heterozygous (*Ffar3*^+/*−*^) was kindly supplied by Dr. Masashi Yanagisawa, University of Texas Southwestern Medical Center^6^. These mice were maintained in a C57BL/6 background. Heterozygous mice were crossed to obtain the global knockout mice (*Ffar3*^−/−^) and genotyped as previously reported^6^. Wild-type littermates (*Ffar3*^+/+^), were used as controls.

Similarly, *Ffar3*-mRFP transgenic mouse line (*Ffar3*^+/*mRFP*^) was kindly donated by Dr. Stefan Offermanns from Max-Planck Institute for Heart and Lung Research, Bad Nauheim, Germany^16^. In this mouse line, the FFAR3 coding exon was replaced with a monomeric red fluorescent protein coding region, which would produce a non-membrane targeted red fluorescent protein. Hemizygous animals were kept on a C57BL/6 background and germline transmission was confirmed by PCR using the following primers set 5′-GGACAACACCGAGGACGTC and 5′ CGTGCTCGTACTGCTCCACC. In addition, *Ffar3*^+/*mRFP*^ hemizygous male animals were bred with female heterozygous *Ffar3*^−/+^ mice in order to generate a nullizygote (*Ffar3*^*−/mRFP*^).

All experiments were performed on adult female/male mice (PND28–84), unless otherwise stated. Animals were maintained on a 12 h day/night cycle with normal chow and water provided *ad libitum*. All procedures involving animals were approved by the Institutional Animal Care and Use Committee (IACUC) of the National Institute on Alcohol Abuse and Alcoholism. Animals were treated, and studies were conducted in accordance with the National Institute of Health Animal Welfare guidelines.

### Quantitative real-time PCR (qPCR)

Female and male mice (PND0-PND365, unless otherwise stated), were anesthetized by CO_2_ inhalation and decapitated before dissection. The tissue/ganglia were rapidly collected in ice-cold Hank’s balanced salt solution, for further cleaning. Then samples were immediately transferred to RNA*later*™ stabilization solution and processed directly or stored at 4 °C overnight. Total RNA was extracted from ganglia/tissue with RNeasy Mini kit, in accordance with the manufacturer’s instructions. RNA quality/quantity was monitored using RNA 6000 Nano LabChip kit on an Agilent Bioanalyzer 2100^66^. Samples with an RNA integrity number (RIN) >8 were used for further experiments. Afterward, 0.8–1 ug of RNA was reverse transcribed using QuantiTec® Reverse transcription kit, as per manufacturer’s instructions. Prior to use in qPCR cDNA was diluted 1:5 with RNase/DNase free water.

Quantitative RT-PCR (qPCR) was performed in triplicate in 20 µl reaction with a StepOnePlus™ Real-time System, following manufacturer’s instructions. Reactions contained TaqMan gene Expression Master mix, TaqMan Primer-probe set (see below), and cDNA according to manufacturer’s protocol. In each reaction 1 µl (Supplementary Fig. 1b,c) and 2 µl (Supplementary Fig. 1a) of cDNA was used per reaction. A custom made *mFfar3* (Ref. Seq. NM_001033316.2; Thermo Fisher Scientific, Waltham, MA, USA, Cat. No. 4441114) primer-probe set was designed to produce an amplicon size of 74 nt. The target position is located at nucleotide 50 and the probe spanned an exon-exon junction, eliminating interference from potential genomic DNA contamination. m*Actb* (4352341E) and m*Gapdh* (4352339E) were used as reference genes. Additionally, each run had samples with the reverse transcriptase omitted as well as master mix alone as negative controls. The mixture was initially activated by heating at 95 °C for 10 min followed by 40 cycles of denaturation steps at 95 °C for 15 s and annealing/elongation at 60 °C for 1 min.

qPCR data were analyzed using a baseline set as per manufacturer’s instructions and thresholds set to 0.1, before being transferred to Excel for quantification of the geometric means. For quantification of relative *Ffar3* gene expression the geometric mean of two internal controls was calculated^67^ and subtracted from CT(*Ffar3*), as follow:

1 $${{\rm{\Delta }}C}_{{\rm{T}}}={\rm{CT}}({\rm{Ffar}}3)\,\mbox{--}\,\sqrt{{\rm{CT}}({\rm{Actb}})\ast {\rm{CT}}({\rm{Gapdh}})}$$

Graphs and statistics were made in GraphPad Prism 7.

### Immunofluorescence (IF)

Mice were transcardially perfused with 20 ml heparinized phosphate buffered saline (0.1 M PBS + 20 units/ml of heparin) and 4% paraformaldehyde (PFA) in 0.1 M PBS, pH 7.4. Ganglia were postfixed overnight in the same fixative at 4 °C and transferred to a 30% sucrose solution and stored at 4 °C until tissues sank into the bottom of the container. Tissue was immersed in Surgipath® FSC22 frozen section embedding medium (Leica Biosystems), and 30 µm longitudinal sections were cut using a Microm HM-500 Cryostat. Cryosections were rehydrated in PBS and treated with 1% Sodium Borohydride for 30 min at room temperature (RT), gently shaking^68,69^. Sections were washed three times with PBS, before being submerged in 0.2% Sudan Black for 1 h^70^. An antigen retrieval procedure was done by incubating sections in 10 mM Sodium Citrate, 100 mM NaCl, pH 6.0 for 30 min. at 80 °C. Next, sections were washed three times with PBS, permeabilized with 0.3% Triton-X 100 for 25 min. at RT, washed three times with PBS and blocked for 1 h at RT. The blocking solution consisted of 2% normal goat serum (NGS) and 2% BSA in Tris-buffered saline with 0.05% Tween 20 (TBS-T, 10 mM Tris base, 200 mM NaCl, pH 7.5). Following blocking, a rabbit polyclonal anti-FFAR3 antibody (1 μg/ml) diluted in blocking solution, was applied to sections overnight at 4 °C. This antibody raised against rat C-terminus FFAR3 sequence (NM_001108912)^8^, was kindly provided by Dr. Jonathan D. Kaunitz and also obtained from Frontier Institute co., ltd., Hokkaido, Japan (RRID: AB_2571697). The peptide sequence has a 92.9% homology with the mouse *Ffar3*, because only two amino acids alanine and glutamic acid, were exchanged with threonine and glutamine, respectively.

Sections were washed in TBS-T for 4 days. Then, the following primary antibodies were used: rabbit polyclonal anti-P2X2 antibody (ab48864, RRID: AB_881833, Abcam, diluted 1:400), mouse monoclonal anti-Calbindin D-28k (CB300, RRID: AB_10000347, Swant® Swiss Antibodies, diluted 1:2500), Chicken polyclonal anti-Neurofilament 200 (NFH, RRID: AB: AB_2313552, Aves Labs, INC., diluted 1:10000), Chicken polyclonal anti-TH (THY, RRID: AB_100113440, Aves Labs, INC., diluted 1:100), Rat monoclonal Anti-Substance P (SP, ab7340, RRID: AB_305866, Abcam, diluted 1:1000) and rabbit polyclonal Anti-Neuropeptide Y (22940, RRID: AB_2307354, ImmunoStar Inc, diluted 1:1000). The specificity of the primary antibodies has been published elsewhere^71–75^. For slices treated with mouse monoclonal CB300 antibody, an additional incubation with unconjugated Goat anti-mouse Fab fragment (10 ug/ml) in blocking solution was applied for 1 h at RT. Slices were washed thoroughly with TBS-T before incubation with the relevant secondary antibody (Alexa Fluor® 488 goat anti-rabbit, A11034; Alexa Fluor® 568 goat anti-rabbit, A11036, Alexa Fluor® 568 goat anti-rat, Alexa Fluor® 633 goat anti-chicken, Alexa Fluor® 405 goat anti-rabbit, Alexa Fluor® 405 goat anti-mouse, all at 1:3000 dilution, Invitrogen). A final TBS-T wash for 1 h at RT was done before an exchange with PBS. Sections were air dried, mounted on slices with Fluoromount-G and coverslipped with No. 1.5 cover glass. Image acquisitions were done with a confocal microscope LSM 880, equipped with DPSS and Argon/Krypton lasers for 405, 488, 561 nm and 603 nm excitation, respectively. Images were acquired with a Carl Zeiss Plan-Apochromat 20x (0.8 NA; FWD = 0.55) in Supplementary Fig. 3 and a 40X water-immersion (1.2 NA; FWD = 0.28) was used on the rest of the images. Optical sections between 1–2 μm and a pinhole adjusted to 1.0–2.0 AU were used for acquisitions. IF in each case was repeated twice. All secondary antibodies were tested for non-specific immunoreactivity by omitting the primary antibodies and no staining was seen.

Fiji software^76^ was used to adjust the contrast of images, cell counting on Figs 2, 4, 5 and Supplementary Figs 4, 5 and to process images on Fig. 2 with a Gaussian blur (sigma = 1.2). Consistently, ganglia from 4 mice were combined and IF was done. In order to determine the labelling index of FFAR3-expressing neurons (mRFP reporter mice and FFAR3 antibody) in Fig. 2, positively stained neurons were scored (0 or 1), counted and normalized to the total number of nuclei within each image. DAPI (diluted 1:10000; Invitrogen Carlsbad, CA) labels all cells present within the ganglia (neurons, Schwann cells, SIF-like cells and satellite glial cells). Data from 8 images were included in the quantification of the percentages of FFAR3-expressing neurons. Likewise, 17 images from 8 animals were analyzed and included in the quadruple labeling studies. In Figs 4, 5 and Supplementary Figs 4, 5 neurons were scored and output was used to generate four-set Venn diagrams using the Venny 2.1 online tool^77^.

### Acute isolation of SCG and CSMG neurons

Male and female young adult and adult mice (PND21-PND84) wild-type, *Ffar3*^*−/mRFP*^ and *Ffar3*^−/−^, were anesthetized by CO_2_ inhalation and decapitated before dissection. SCG/CSMG were dissected and neurons dissociated in accordance with Won *et al*. 2013. Neurons were maintained in humidified 95% air/5% CO_2_ incubator at 37 °C. Electrophysiological recordings were done the same day after neurons isolation.

### Electrophysiology

Dissociated SCG/CSMG and mRFP positive neurons were identified with a Nikon Diaphot microscope with an X-Cite® 120 PC Q fluorescence illumination system and were voltage-clamped with the conventional whole-cell configuration patch-clamp technique^78^. Pipettes were pulled from Schott 8250 glass (King Precision Glass, Claremont, CA) on a Sutter P-97 puller. Then they were coated with silicone elastomer (Sylgard 184, Dow Corning, Midland, MI) and fire-polished using an in-house made microforge (MF-830, Narishige, Tokyo, Japan). After fire-polishing, the pipette resistance was 1.5–2 MΩ, when filled with the internal solution detailed below. All recordings were made at room temperature and the bath was grounded using a 0.13 M KCl/agar salt bridge, connecting an Ag/AgCl pellet to the bath solution.

Ca^2+^ currents were recorded using an Axopatch 200B series amplifier and digitized with an ITC-18 data acquisition interface (InstruTECH, Port Washington, NY). Experiments were controlled by a Macintosh computer, running S6 data acquisition software written by Dr. Stephen Ikeda (NIAAA/NIH, Bethesda, MD). Pipette capacitance was fully compensated while in the cell-attached configuration. When whole-cell configuration was reached, a −10 mV pulse was applied from a membrane potential of −80 mV. Capacitance transients were recorded and the decay time, series resistance and membrane capacitance were estimated. Then, cell membrane capacitance was canceled, and series resistance was compensated at 90% prediction and correction with a lag of 5 μs. The average series resistance of recordings was 6.37 ± 0.2 MΩ (n = 74). This typically resulted in a calculated transmembrane voltage errors <2 mV at peak density current (−16.88 ± 0.75 pA/pF). Calcium current traces were acquired at 10 kHz with a 16-bit analog-to-digital converter board (ITC-18, HEKA, Bellmore, NY), filtered at 2 kHz (−3 dB; 4-pole Bessel) and stored on the computer for later analyses.

Modulation of N-type Ca^2+^-channel following G protein activation was measured with a double-pulse protocol as stated in Figs 1a and 3a. This protocol was applied before and after the addition of 1 mM sodium propionate (C3, P1880, Millipore-Sigma, St Louis, MO). The percentage of calcium current (I_Ca_^2+^) prepulse inhibition was calculated using the current amplitude measured isochronally 10 ms after the onset of the test pulse and following the equation:

2 $$({{\rm{I}}}_{{\rm{control}}}-{{\rm{I}}}_{{\rm{C}}3}/{{\rm{I}}}_{{\rm{control}}})\ast 100$$

Commonly, basal facilitation ratio is defined as the ratio of isochronal Ca^2+^ current amplitudes elicited after (postpulse) and before (prepulse) a strong depolarizing voltage step, in the absence/presence of C3.

For I-V relationship, the membrane potential was held for 80 ms from −120 mV to +80 mV (in a 5 to 10 mV increments), from a holding potential of −80 mV as shown in Supplementary Fig. 2b. The peak currents were normalized to whole-cell membrane capacitance, recorded before each experiment.

The extracellular solution was (in mM): 140 methanesulfonic acid, 145 tetraethylammonium hydroxide (TEA-OH), 10 HEPES, 10 glucose, 10 CaCl_2_ and 0.0003 tetrodotoxin, pH 7.4 with TEA-OH. All experiments were carried out at 20–25 °C. Patch pipettes were filled with an internal solution containing (in mM) 120 N-methyl-d-glucamine, 20 TEA-OH, 11 EGTA, 10 HEPES, 10 sucrose, 1 CaCl_2_, 14 Tris-creatine phosphate, 4 MgATP and 0.3 Na_2_GTP, pH 7.2 with methanesulfonic acid.

Drug solutions were applied directly to neurons using a custom-made gravity perfusion system. All Sodium propionate (C_3_) solutions were diluted to a final concentration of 1 mM, from a freshly made 1 M stock solution and pH was checked/adjusted to pH 7.4–7.45.

### Statistics

Statistical analyses were performed using Prism 7 (GraphPad Software, La Jolla, CA). Welch’s *t*-test was used with heteroscedastic data (differences in standard deviations between groups), and with unequal/small sample sizes. A *t*-test was used when all the assumptions were met. D’Agostino & Pearson omnibus K2 normality test (α = 0.05) was used to test data deviation from Gaussian ideal. Likewise, Kruskal-Wallis *H* test followed by Dunn’s multiple comparisons test was used for nonparametric data. Values were expressed as mean ± standard error (S.E.M.), unless otherwise noted. Values non-significant were indicated by n.s.

## Electronic supplementary material

Supplementary Information

## Data Availability

Specific material, methods, raw/analyzed data in the current study are available by contacting the corresponding author.
