# Supplementary material for: Selective tracking of FFAR3-expressing neurons supports receptor coupling to N-type calcium channels in mouse sympathetic neurons
Source: Sci Rep. 2018 Nov 26;8:17379. doi: 10.1038/s41598-018-35690-z (PMC6255804; doi:10.1038/s41598-018-35690-z)
Supplement: Supplementary file 1 — Supplementary Information [file 41598_2018_35690_MOESM1_ESM.pdf]

Selective tracking of FFAR3-expressing neurons supports receptor coupling to N-type calcium channels in mouse sympathetic neurons.

Claudia Colina[1*], Henry L. Puhl III[2] and Stephen R. Ikeda[2].

Supplementary Figures:

Figures 1

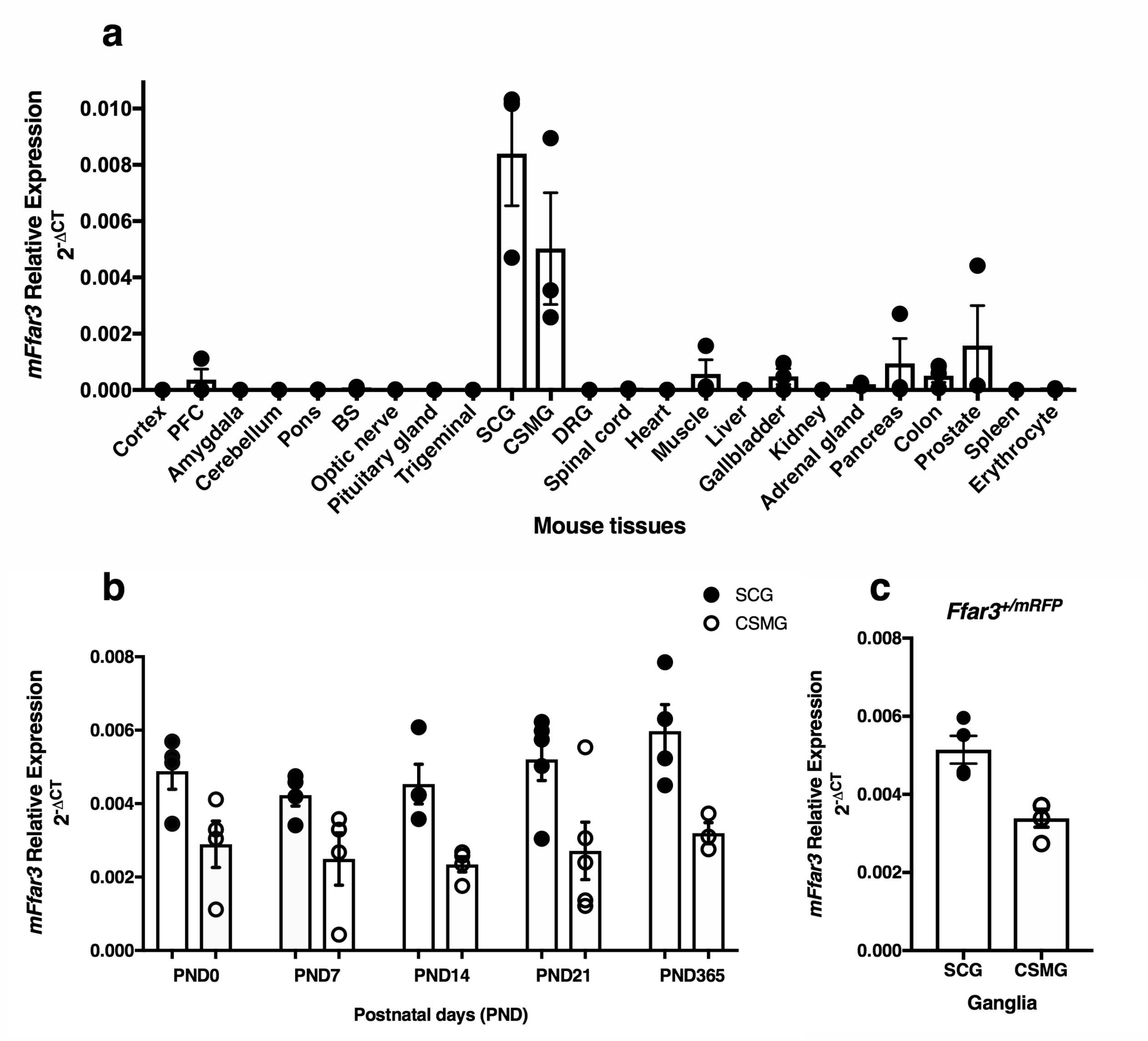

Figures 2

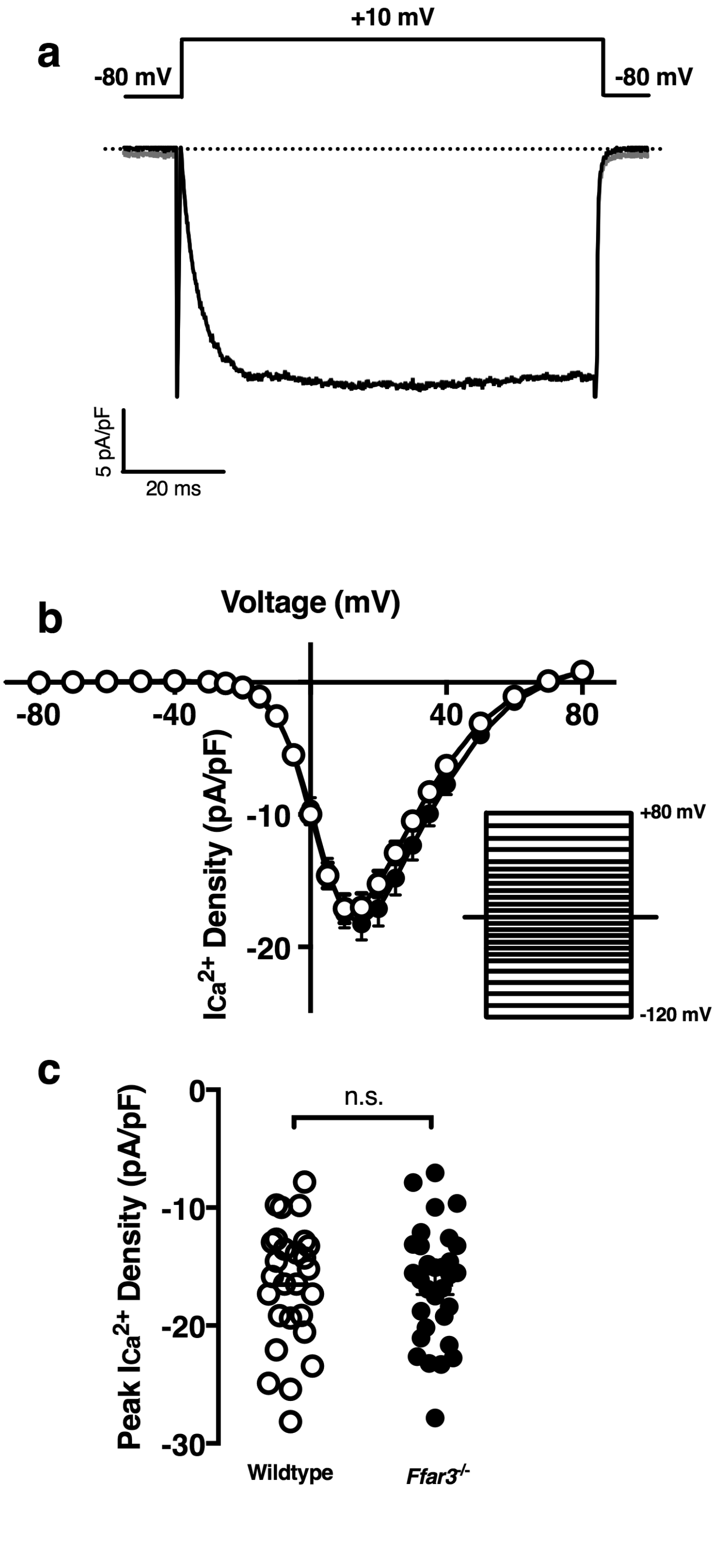

a
+10 mV
-80 mV
-80 mV
5 pA/pF
20 ms
b
Voltage (mV)
-80
-40
40
80
-10
-20
Ica2+ Density (pA/pF)
+80 mV
-120 mV
c
Peak Ica2+ Density (pA/pF)
0
-10
-20
-30
n.s.
Wildtype
Ffar3-/-

Figures 3

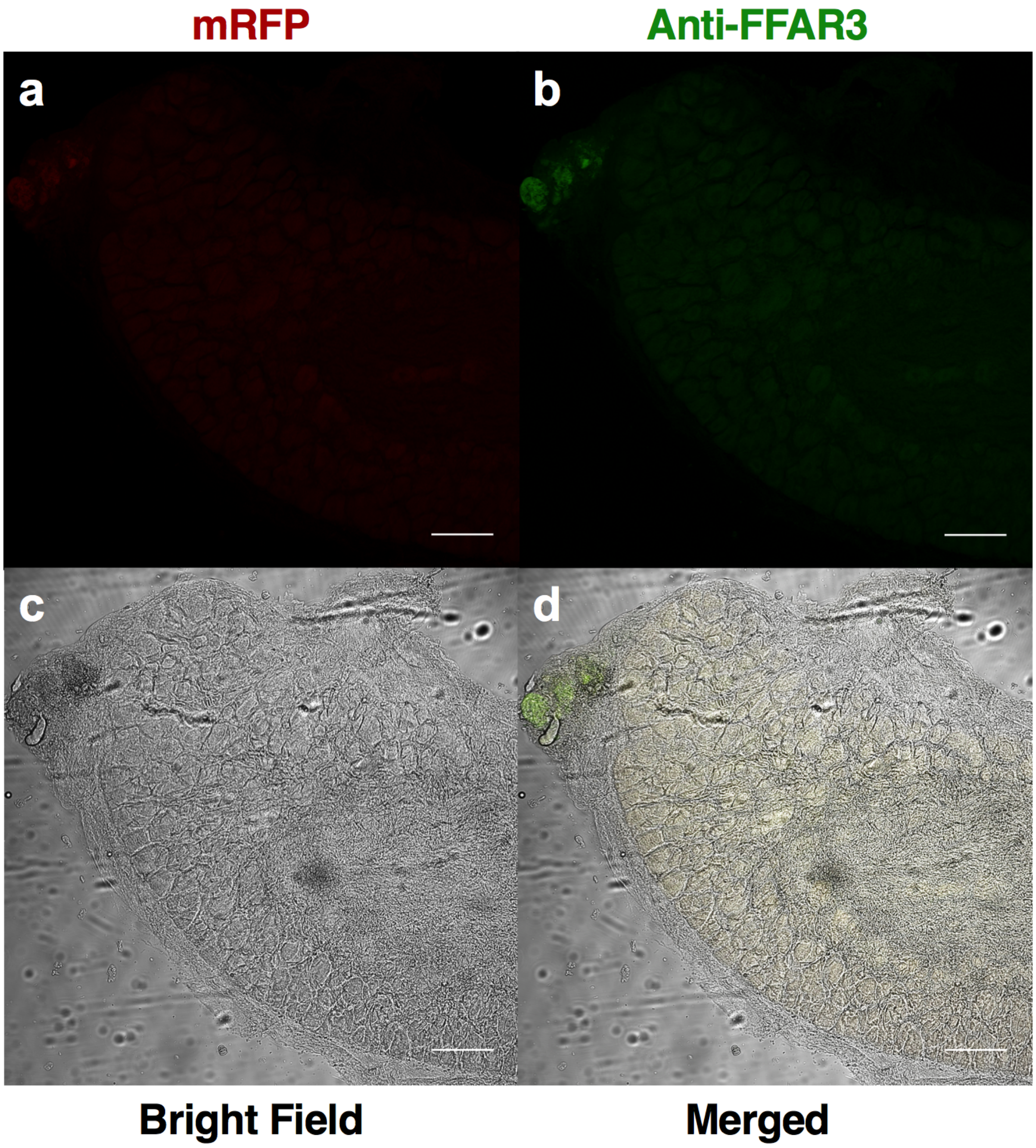

Figures 4

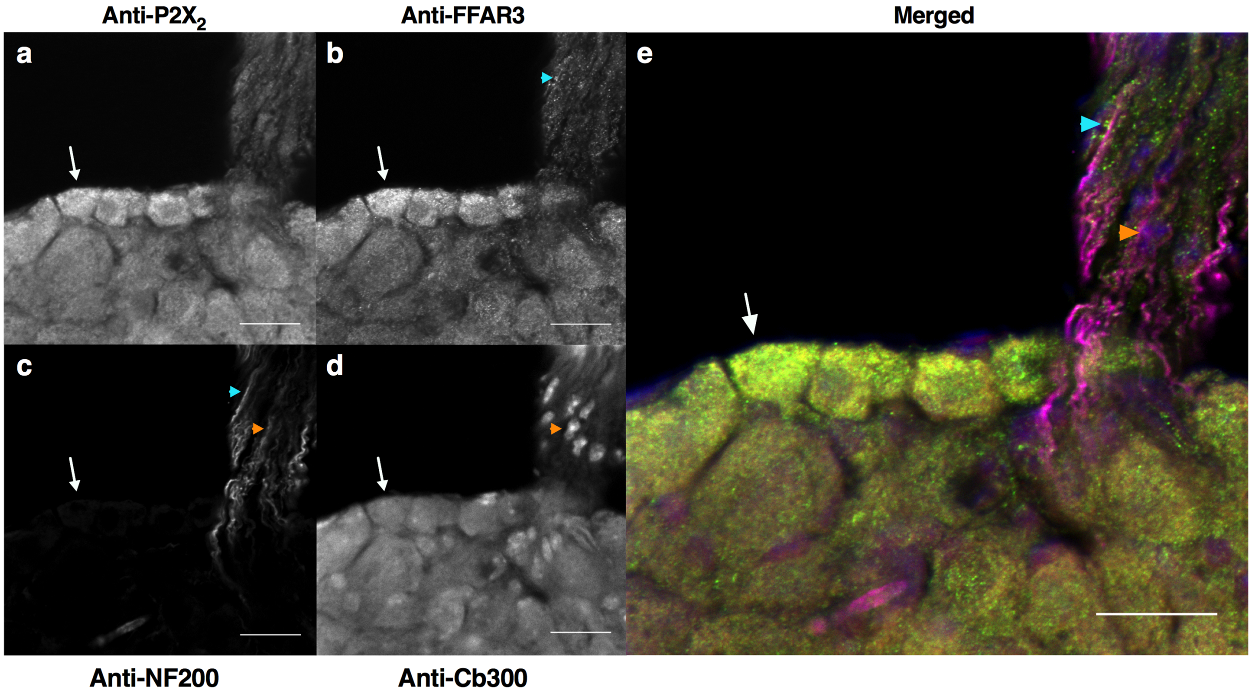

Figures 5

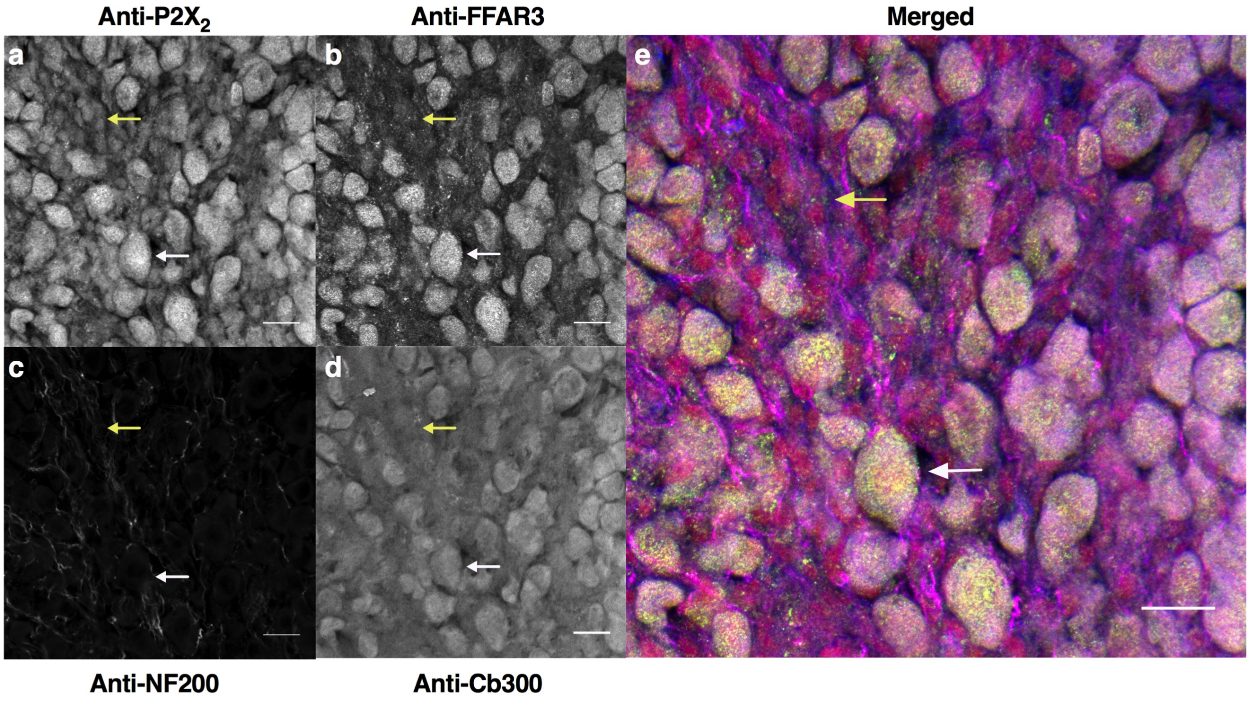

Supplementary Figures legends.

Supplementary Figure 1: ***Ffar3* gene expression in mice tissues, developmental dynamic changes and *Ffar3* gene expression in mRFP hemizygous mice in pre/paravertebral ganglia.**

a. Postnatal expression (PND 56-84) of *Ffar3* mRNA in different wild-type mouse tissues. PFC=prefrontal cortex, BS=Brainstem, SCG=superior cervical ganglion, CSMG=celiac-superior mesenteric ganglion, DRG=dorsal root ganglion. Total RNA was isolated from the indicated tissue/ganglia at postnatal day PND 84-168 (n = 3) from 8 mice and cDNA was reverse transcribed as indicated in materials and methods. A stochastic dominance was found on every tissue when compared to SCG *Ffar3* gene expression Kruskal-Wallis test followed by Dunn's multiple comparisons test (SCG vs different tissues ***, KW=55.6, α=0.05 and p=0.0002). b. Postnatal expression of *Ffar3* mRNA in SCG (black circles) and CSMG (white circles). Total RNA was isolated from the indicated ganglia at postnatal day (PND) 0 (n = 4), 7 (n = 4), 14 (SCG n = 3, CSMG n=4), 21(n = 5), and 365 (n = 3). No evidence of stochastic dominance was found with the Kruskal-Wallis test followed by Dunn's multiple comparisons test (SCG KW= 5.16, p=0.27 and CSMG KW= 2.99, p=0.56). c. Postnatal expression of *Ffar3* mRNA in SCG (black circles) and CSMG (white circles) in $Ffar3^{+/mRFP}$ hemizygous mice. Total RNA was isolated from the indicated ganglia at postnatal day (PND) 182 (n = 4). In a, b and c *Ffar3* mRNA was quantified by qPCR using a custom made TagMan primers-probe set, targeted to the nucleotide 50 and probe was positioned to span an exon-exon junction. *Ffar3* gene expression was normalized to the geometric mean of *Actb* and *Gapdh* housekeeping genes and expressed as *Ffar3* relative expression.

Figure 2: **Current densities are not altered by gene ablation of *Ffar3*.**

a. Representative $I_{Ca}{}^{2+}$ density time courses for wild-type (black line) and $Ffar3^{-/-}$ (red line). Voltage step is shown in the inset of panel a, 100 ms pulse from -120 mV to +80 mV. Only the $I_{Ca}{}^{2+}$ density generated by a +10 mV voltage step is showed for clarity. In order to obtain the $I_{Ca}{}^{2+}$ density, currents were normalized to whole-cell membrane capacitance (Cm). Cm was calculated by integration of an uncompensated cell capacitive transient current (charge, Q) elicited by 10mV depolarizing test pulse for 5 ms, from a holding potential of -80 mV and using

the equation Cm=Q/V (3). b. Mean I_{Ca}^{2+} density (pA/pF)-voltage (mV) relations. I_{Ca}^{2+} was evoked from a holding potential of -80 mV to the test potential indicated in inset of panel b. I_{Ca}^{2+} amplitudes and normalization was done as described in a. Each point represents mean ± S.E.M. of wild-type neurons (open circles, n=25) and knockout mice (*Ffar3*$^{-/-}$, black circles, n=27). Points from -120 to -90 mV have been omitted for clarity. c. Peak current densities at +10 mV calculated from current recordings using acutely dissociated CSMG neurons from wild-type and *Ffar3*$^{-/-}$. Two-tailed unpaired *t*-test was used to determine whether the difference between the population means (wild-type vs *Ffar3*$^{-/-}$) were statistically significant. The difference between the means was not statistically significant, t(56)=0.016, with α= 0.1, 90% CI: -2.2-2.2, n_1=27, n_2=31 and *p*=0.99. Four wild-type animals and three knockout mice were used to generate the plots.

Figure 3: **Cryosections of *Ffar3*-reporter hemizygous mice dorsal root ganglion (DRG) FFAR3-labelled used as a control.**

Representative fluorescent IF images of three repetitions with DRG cryosections from hemizygous *Ffar3*$^{+/mRFP}$ reporter mice. Left upper panel: Direct mRFP (red, a) fluorescence detection in DRG slices counterstained with Anti-FFAR3 antibody (green, b). c. Bright field image and d superimposed images. Note that mRFP and FFAR3 antibody fluorescence are not present in this ganglion, which correlates with *Ffar3* mRNA levels. Scale bars are 50 μm and the same in all images.

Figure 4: **Cryosections of mice celiac-mesenteric ganglia quadruple-labelled to show chemical coding of postganglionic neurons and FFAR3-expressing neurons.**

Quadruple-staining of CSMG sections were performed with Anti- $P2X_2$ ionotropic purinergic receptor (Anti- $P2X_2$, a), Anti-FFAR3 (b), Anti-Neurofilament 200 kDa (Anti-NF200, c) and Calbindin D-28k (Anti-Cb300, d). Cyan arrowhead indicates A-fiber surrounded by FFAR3 punctuate immunoreactivity (b-c and e). Orange arrowhead indicates Schwann cells associated with A-fibers (c-e). Labeling was repeated twice and using four wild-type animals on each replicate. Scale bars in all images are 20 μm.

Figure 5: **Cryosections of mice superior cervical ganglia quadruple-labelled to show chemical coding of postganglionic neurons and FFAR3-expressing neurons.**

Quadruple-staining of SCG sections were performed with Anti-$P2X_2$ ionotropic purinergic receptor (Anti-$P2X_2$, a), Anti-FFAR3 (b), Anti-Neurofilament 200 kDa (Anti-NF200, c) and Calbindin D-28k (Anti-Cb300, d). White arrow indicates representative neurons that is $P2X_2$-, FFAR3- and Cb300-IR. Yellow arrow indicates a representative group of SIF-like cells that are $P2X_2$-IR and Cb300-IN, FFAR3-IN. Labeling was repeated twice and using four wild-type animals on each replicate. Scale bars in all images are 20 μm.
